# Supplementary material for: Smoking, alcohol consumption, and cancer: A mendelian randomisation study in UK Biobank and international genetic consortia participants
Source: PLoS Med. 2020 Jul 23;17(7):e1003178. doi: 10.1371/journal.pmed.1003178 (PMC7377370; doi:10.1371/journal.pmed.1003178)
Supplement: Attachment — Submitted filename: Rebuttal letter.docx [file pmed.1003178.s009.docx]

# **Response to Editors’ and Reviewers’ comments**

**Requests from the editors:**

Ref 1 raises important points, which we think are especially important to address.

General point: please tone down sentences such as this in the abstract: “These findings indicate that smoking is causally associated with an increased risk of cancer”. While MR study is indicative, it cannot show causality and so please change this sentence and other instances in the text (including the Author Summary and the conclusion section).

Please provide p values in the abstract with 95% CIs. In addition, is it possible to include some summary demographic information in the abstract?

Please remove spaces in between multiple refs in square brackets.

Please use sections and Paragraphs for the STROBE – pages change during revisions and formatting etc.

**Response: Thank you for these points. As recommended, we have:**

**(1) toned down the sentences about causality in the abstract, Author Summary, and Conclusion section;**

**(2) provided the p-values with 95% CIs in the abstract;**

**(3) provided summary demographic information in the abstract;**

**(4) removed spaces in between multiple refs in square brackets;**

**(5) used sections and paragraphs, instead of page number, in the STROBE checklist.**

-----------------------------------------------

**Comments from the reviewers:**

**Reviewer #1:**

Larsson et al. use Mendelian randomisation (MR) to examine the relationship between smoking and alcohol consumption, and 19 site-specific cancers. The manuscript is concise and well written, and the methodologies applied are appropriate. The key limitation of the study is its low power to identify causal relationships with many of the cancers. This is especially true for alcohol consumption, which the authors causally relate to no site-specific cancer, despite strong prior evidence from other studies. This low power prevents the study from providing further information on cancers for which evidence of a relationship with smoking and alcohol consumption has been so far mixed.

Major comments:

1) Low power to detect causal relationships occurs due to the authors use of UK BioBank data for the site-specific cancers. I think this is the main issue with this study. For many cancers, much larger datasets have been published and are publicly available (for example the BCAC breast cancer GWAS contains >100,000 breast cancer cases, in contrast to the ~14,000 breast cancer cases in UK BioBank). Where possible, these larger cancer GWAS should be used in place of the UK BioBank data. I appreciate that in some instances data access limitations and scientific politics would prevent the authors of this manuscript from accessing these larger data sets. However, use of larger datasets where possible would substantially improve this analysis. Currently, the analysis of alcohol consumption with site-specific cancer risk feels like a missed opportunity, given that for 13/19 site-specific cancer, the study has less than 80% power to detect ORs of 3.

**Response: Thank you for this and the other comments. As recommended, we have added results for genetically-predicted smoking and alcohol consumption in relation to cancer risk based on publicly available data from large-scale GWAS consortia of breast cancer (BCAC), ovarian cancer (OCAC), prostate cancer (PRACTICAL), and lung cancer (ILCCO).**

2) Where UK BioBank data cannot be replaced with data from a larger cancer GWAS, further information should be provided about how cancer association statistics were computed using UK BioBank data. Did the authors compute these statistics themselves, or were they obtained from another source, such as the Neale Lab (who have computed association statistics for UK BioBank data. Were any samples excluded for QC reasons?

**Response: We have computed cancer association statistics ourselves. This enables us to define disease outcomes in a bespoke and comprehensive way which combines information on related ICD 9 and 10 codes. This would not be possible using precomputed association estimates.**

**We have now made this clear in the Methods (first paragraph) and we have added a table describing the sources and definitions of each cancer (S2 Table). The revised text is as follows: “In addition, we estimated genetic associations with 19 site-specific cancers (with at least 400 cases) and overall cancer in UK Biobank, a cohort study of about 500 000 adults (37 to 73 years of age at baseline) recruited between 2006 and 2010 [21]. In the current analyses, we included 367 643 UK Biobank participants of European descent after exclusion of participants with other ethnicities (to reduce population stratification bias), those with relatedness of third degree or higher, excess heterozygosity, and low genotype call rate. Cancer cases were ascertained until March 31, 2017, and were defined based on data from national registries (International Classification of Diseases ninth and tenth revision codes) and self-reported information verified by interview with a nurse (S1 Table). We calculated beta coefficients and standard errors of the genetic associations with cancer using logistic regression, with adjustment for age, sex, and ten genetic principal components.”**

3) The authors note that sample overlap between the exposure and outcome datasets is likely biasing their results. Ideally, the authors should recompute association statistics for smoking and alcohol consumption excluded UK BioBank participants. If this is not possible, then this caveat should be more prominent, preferably in the abstract. The potential magnitude of this bias should also be estimated, as per Burgess et al.

(<https://eur01.safelinks.protection.outlook.com/?url=https%3A%2F%2Fwww.ncbi.nlm.nih.gov%2Fpubmed%2F27625185&amp;data=02%7C01%7Csusanna.larsson%40ki.se%7Cf511bfa114904c0a969408d7c670255a%7Cbff7eef1cf4b4f32be3da1dda043c05d%7C0%7C0%7C637196057272197540&amp;sdata=M6WfzM75QPgS%2FDhJkXmJo53FNwuGFQBlx3zRrIJCilM%3D&amp;reserved=0>).

**Response: We do not have access to individual-level data from the studies included in the meta-analysis of the GWASs of smoking and alcohol and cannot redo those analyses after excluding UK Biobank. However, we have added results based on data from GWAS consortia for breast, ovarian, prostate, and lung cancer which did not include UK Biobank participants, thereby avoiding potential bias due to partial sample overlap.**

**As is typical when genetic variants are associated with the exposure at a genome-wide level of statistical significance, the F statistics for the instruments in the analytic sample are above 10. In combination with the relatively low proportion of sample overlap, bias arising due to sample overlap is relatively small. (It is not simple to present calculations for every exposure--outcome pair as the degree of bias is different for each pair, depending on the number of cases and the observational association between the exposure and outcome. But in each case, bias is low.)**

4) We have previously observed that Wald-type ratio estimators do not provide accurate estimates of the causal OR when both the exposure and outcome traits are binary (https://eur01.safelinks.protection.outlook.com/?url=https%3A%2F%2Fwww.ncbi.nlm.nih.gov%2Fpubmed%2F29540232&amp;data=02%7C01%7Csusanna.larsson%40ki.se%7Cf511bfa114904c0a969408d7c670255a%7Cbff7eef1cf4b4f32be3da1dda043c05d%7C0%7C0%7C637196057272197540&amp;sdata=gEe88K1cZ%2BEfhBHRtG87qrZ5zKWWJ6gLlXj%2FHLKfc%2F8%3D&amp;reserved=0, full disclosure: I am an author on this paper). This has also been noted by others (e.g. Palmer et al.

<https://eur01.safelinks.protection.outlook.com/?url=https%3A%2F%2Fwww.ncbi.nlm.nih.gov%2Fpubmed%2F21555716&amp;data=02%7C01%7Csusanna.larsson%40ki.se%7Cf511bfa114904c0a969408d7c670255a%7Cbff7eef1cf4b4f32be3da1dda043c05d%7C0%7C0%7C637196057272207530&amp;sdata=g5dk6L59bNujZEFD1mINDHhq2rryqc0MJveUhqZa2xA%3D&amp;reserved=0>). Are the causal estimates between smoking initiation and cancer risk reported here potentially affected by such bias? If so, this should be noted.

**Response: For a binary exposure, it is difficult to express a causal effect on a meaningful scale. Typically, estimates would represent a comparison between states representing the presence and absence of the exposure. However, this is problematic for Mendelian randomization analyses, as typically genetic variants do not have such striking effects on risk factors.**

**We have been careful to express all estimates in terms of the association of genetically-predicted levels of the exposure, rather than making claims about the numerical magnitude of the causal effect. The Wald-type methods are unbiased under the null (see Vansteelandt et al, Statistical Science 2011 “On instrumental variables estimation of causal odds ratios”), and so they provide a valid test of the causal null hypothesis even if the estimates do not reflect meaningful causal parameters.**

Minor comments:

1) Supplementary Figure 1 should be annotated with the site-specific cancers, to make it easier for readers to identify the cancers for which the study has suitable power.

**Response: We have replaced Supplementary Figure 1 with a table showing the power to detect different odds ratios for each cancer site (S2 Table).**

2) The authors consider their analysis of overall cancer risk to be their "primary" analysis, whilst the analysis of site-specific cancers are "secondary" analyses. How smoking affects cancer risk differs substantially between sites (as seen in Figure 2), and I am therefore unsure how useful the analysis of overall cancer risk is. The analysis of site-specific cancers is potentially more interesting, and therefore this should be the "primary" analysis.

**Response: We agree that cancer is a heterogeneous set of diseases, but results for smoking and alcohol consumption in relation to overall cancer are relevant from a public health perspective. Nonetheless, following this comment, we have toned down the overall cancer results by removing the figure for overall cancer and presented the overall cancer results in the supplement (S3 and S5 Tables) and last paragraph of the Results section. In addition, the overall cancer results are considered complementary analyses (Introduction, last sentence). We have added a section in the discussion on the limitations of analyses for overall cancer.**

3) The conclusion currently states: "These MR findings indicate smoking is causally associated with an increased risk of cancer, particularly of the lung...". The causal association between smoking and lung cancer is very well understood and more focus should therefore be placed on the site-specific cancers over which there is more debate.

**Response: As recommended, we have modified the conclusion. The revised conclusion about smoking and cancer is as follows (Abstract and Conclusions of the main text):**

**“This study confirms the well-established relationship between smoking and lung cancer, and indicates that smoking may also be a risk factor for cancer of the head and neck, oesophagus, stomach, cervix, and bladder.”**

4) Further information should be provided about how the lifetime smoking IV takes into account duration, heaviness etc.

**Response: Due to the weaker instrument for lifetime smoking (compared with smoking initiation), the complete sample overlap with UK Biobank, and the difficulty to interpret the results based on this instrument (consisting of a combination of smoking status, duration and heaviness), we have removed this supplementary analysis for the lifetime smoking index.**

5) The authors apply Bonferroni correction to overall cancer risk, but not the site-specific cancers. Multiple testing should be corrected for in both analyses.

**Response: As recommended we have applied multiple testing for the site-specific cancers. The new p value threshold is 0.0013 (Statistical analysis).**

--------------------------------

**Reviewer #2:**

This is a two-sample MR analysis that investigates the association of smoking initiation, lifetime smoking and alcohol consumption with several cancers in the UK Biobank. This is the largest MR study investigating smoking, alcohol in relation to cancer risk. It has verified several positive associations observed in the observational literature for smoking and risk of several cancers, but failed to do so for alcohol consumption; the authors have correctly reasoned this finding to the low power of the alcohol consumption analysis, as only 0.3% of the variation in alcohol consumption is explained by the known GWAS identified genetic variants. The analysis is straightforward and the paper is well-written. I have only a few minor comments:

1.  In the Introduction, second paragraph, where the authors describe the alcohol and cancer observational literature, they should add that the evidence is also strong for high alcohol consumption and risk of stomach and colorectal cancer.

**Response: Thank you for this and the other comments. We have added that the evidence is also strong for heavy alcohol consumption and risk of stomach and colorectal cancer (Introduction, second paragraph).**

2.  The authors performed an analysis for lifetime smoking using a UK Biobank GWAS to define relevant instruments. They have already commented in the Discussion about potential bias caused due to overlap of the exposure and outcome datasets. It would be nice to extend the discussion to situations where the overlap is complete.

**Response: Following this comment and due to the much weaker instrument for lifetime smoking (compared with smoking initiation), the complete sample overlap with UK Biobank, and the difficulty to interpret the results based on this instrument, we have removed this supplementary analysis for the lifetime smoking index.**

3. Cancer is a heterogeneous set of diseases, and it doesn't make a lot of sense to conduct analyses for all cancer sites combined.

**Response: We agree that cancer is a heterogeneous set of diseases, but results for smoking and alcohol consumption in relation to overall cancer are relevant from a public health perspective. Nonetheless, following this comment, we have toned down the overall cancer results by removing the figure for overall cancer and presented the overall cancer results in the supplement (S3 and S5 Tables) and last paragraph of the Results section. In addition, the overall cancer results are considered complementary analyses (Introduction, last sentence). We have added a section in the discussion on the limitations of analyses for overall cancer.**

4.   The contamination mixture method developed by one of the study authors could be another method that the investigators could use for smoking initiation to investigate potential pleiotropy but also whether the 361 SNP IV could be subgrouped to more than one potential mechanisms.

**Response: We have added results based on the contamination mixture method (S3 Table and S5 Table).**

---------------------------

**Reviewer #3:**

This study uses the approach of Mendelian randomization (MR) to assess causality for associations of cancer with smoking and alcohol consumption, respectively. While this is an interesting and worthwhile study and the methodology seems to be sound, I feel that the authors somewhat overestimate the explanatory power of their study. Given that causality has already been established for many of the associations based on comprehensive reviews of experimental and observational evidence by renowned institutions (International Agency for Research on Cancer, US Surgeon General, World Cancer Research Fund…), it seems weird when the authors question causality of associations to justify their MR approach, even though they can only provide low precision estimates due to the small amount of variance explained by their genetic instruments despite high sample sizes and thus cannot exclude false-negative findings.

Having said that, their approach is indeed worthwhile and could be used to further underpin available evidence. But I nevertheless would recommend authors to tone down some of their statements. For example on page 9 of the discussion, where authors state that their findings "extend the observational evidence that smoking is also a risk factor for cancers of the head and neck, oesophagus, stomach, cervix, and bladder." The causality of these associations has been already well-established for years and decades. The authors go on to say that their results offer "strong support for smoking to be considered as a risk factor for this wider range of cancers in clinical practice". I think we are already way past this point - the long available evidence should have already been translated into clinical practice years ago.

**Response: Thank you for the comments. As recommended, we have toned down the corresponding statements. We have removed the sentence “Taken together, this offers strong support for smoking to be considered as a risk factor for these cancers in clinical practice.”**

The study in its current form is missing one important piece of the puzzle: there is no data supporting whether in this specific sample the genetic instruments are actually associated with higher smoking and alcohol consumption, respectively. This would be further confirmation for the suitability of the genetic instruments, but it would also allow for better assessment of the statistical power to detect effects. Some information on the amount of variance explained by the genetic instruments is reported in the manuscript, but my understanding is that those stem from published meta-analyses. I would thus recommend to add some data on the associations between the genetic instruments and different indicators of smoking and alcohol exposure in the sample.

**Response: We have tested the genetic associations with alcohol consumption and smoking behaviour in the UK Biobank. The F-statistics of the genetic instruments provided in the paper are based on our own analyses in the study population (UK Biobank). This is an important measure of the overall strength of association of the variants with the exposure as it is relevant to judging the likely degree of bias due to weak instruments and sample overlap. We have now clarified this in the paper (page 7). We have modified the sentence about the genetic instruments as follows (page 7):**

**“The genetic instruments were strongly associated with the exposures in UK Biobank, with an F statistic from regression of the exposure on the variants of about 75 for smoking initiation and 19-29 for alcohol consumption.”**
