# Supplementary material for: Smoking, alcohol consumption, and cancer: A mendelian randomisation study in UK Biobank and international genetic consortia participants
Source: PLoS Med. 2020 Jul 23;17(7):e1003178. doi: 10.1371/journal.pmed.1003178 (PMC7377370; doi:10.1371/journal.pmed.1003178)
Supplement: Attachment — Submitted filename: Response to Editors 2nd revision.docx [file pmed.1003178.s010.docx]

**Requests from Editors:**

1. Response to Reviewer comments: As requested by the reviewer, please include your caveat regarding Reviewer 1, Point #4, a discussion point in the manuscript (perhaps in the section describing limitations).

**Response: Following the comment from Reviewer 1, Point #4, the following text has been added to the limitations (page 16):**

**“An additional limitation is that smoking initiation is a binary exposure. An MR estimate with a binary exposure and binary outcome is difficult to interpret as a specific causal effect [42]. Furthermore, the MR estimate cannot simply be compared with the association between self-reported smoking and cancer risk estimated by observational studies. However, even in this setting, MR estimates are unbiased under the null [43], and thus provide a valid test of the causal null hypothesis even if the estimates do not reflect meaningful causal parameters. Here, we expressed all estimates in terms of the association between genetically-predicted levels of smoking initiation and the outcome, rather than making claims about the numerical magnitude of the causal effect.”**

2. Title: Please mention the study population in the title (e.g. the UK Biobank, consortia).  Please revise your title, we suggest: “Smoking, alcohol consumption and risk of cancer: A Mendelian randomisation study in UK Biobank and international genetic consortia participants” or similar.

**Response: We have revised the title as suggested. Thank you for all the great suggestions for improvement of the manuscript. Very appreciated.**

3. Competing Interests: Please add this statement to the manuscript's Competing Interests in the manuscript submission form: "SB is a paid statistical consultant on PLOS Medicine's statistical board."

**Response: The statement has been added to the Competing Interests in the manuscript submission form.**

4. Data Availability: Thank you for providing the summary-level data as a supporting information file.  However, we require that you please make the de-identified primary data available, or provide contact information for interested researchers to apply for access to such data (please note that the contact for data access cannot be one of the study’s authors).

**Response: In addition to providing the summary-level data supporting the results presented in this study (S1 Data file), we have added the following statement in the Data Availability section: “Primary data from the UK Biobank study are accessible upon application (**[**https://www.ukbiobank.ac.uk/)**](https://www.ukbiobank.ac.uk/))**.” We are unable to make de-identified primary data available ourselves, but such data are available on application to any *bona fide* scientific researcher.**

PLOS defines the “minimal data set” to consist of the data set used to reach the conclusions drawn in the manuscript with related metadata and methods, and any additional data required to replicate the reported study findings in their entirety. Authors do not need to submit their entire data set, or the raw data collected during an investigation. Please submit the following data:

The values behind the means, standard deviations and other measures reported;

The values used to build graphs;

The points extracted from images for analysis.

**Response: The values used to build graphs and points extracted from images have been added in the S1 Data file. Values behind the other measures are also provided in this file. No mean values were calculated. The data provided are sufficient to reproduce all analyses presented in the data.**

5. Prospective analysis plan: Did your study have a prospective protocol or analysis plan? Please state this (either way) early in the Methods section.

a) If a prospective analysis plan (from your funding proposal, IRB or other ethics committee submission, study protocol, or other planning document written before analyzing the data) was used in designing the study, please include the relevant prospectively written document with your revised manuscript as a Supporting Information file to be published alongside your study, and cite it in the Methods section. A legend for this file should be included at the end of your manuscript.

b) If no such document exists, please make sure that the Methods section transparently describes when analyses were planned, and when/why any data-driven changes to analyses took place. 

c) In either case, changes in the analysis-- including those made in response to peer review comments-- should be identified as such in the Methods section of the paper, with rationale.

**Response: We have added the following paragraph (page 9):**

**“Protocol**

**There was no formal predefined protocol or prospective analysis plan for this study. The MR analyses of genetic predisposition to smoking and alcohol consumption in relation to overall and site-specific cancers in UK Biobank were initiated in August 2019. Following peer review, we (i) conducted MR analyses of smoking and alcohol consumption in relation to lung, breast, ovarian, and prostate cancer using publicly available data from international consortia for these cancers; (ii) defined the analysis of site-specific cancer as the primary analysis and applied Bonferroni correction to site-specific cancers; (iii) calculated statistical power for each site-specific cancer; and (iv) performed a complementary analysis using the contamination mixture method.”**

6. Abstract (and throughout manuscript): Early in the abstract, the wording could be adjusted to acknowledge that there is, in fact, little or no doubt that smoking causes lung cancer. Also, and more generally throughout the paper, the wording should accommodate existing knowledge more realistically (e.g., the ACS website states quite unambiguously that "Smoking is the most important risk factor for bladder cancer. Smokers are at least 3 times as likely to get bladder cancer as non-smokers. Smoking causes about half of all bladder cancers in both men and women.") Perhaps "genetic predisposition to smoking" is an imperfect proxy for actual smoking.

**Response: We have revised the first two sentences of the abstract to: “Smoking is a well-established cause of lung cancer and there is strong evidence that smoking also increases the risk of several other cancers. Alcohol consumption has been inconsistently associated with cancer risk in observational studies.”**

**In the Discussion, we have added the following sentence (page 13): “Smoking is the major risk factor for bladder cancer and it has been estimated that ever smoking accounts for about two-thirds and one-thirds of all bladder cancer cases in men and women, respectively [6].”**

7. Abstract: Introduction: Please revise the final sentence to: “Mendelian randomisation study sought to investigate associations in support of a causal relationship between smoking and alcohol consumption and 19 site-specific cancers.”

R**esponse: We have amended the sentence as suggested.**

8. Abstract: Methods and Findings: Please identify some demographics of the participants included in the study (country, etc.) for the genome-wide association studies consortia.

**Response: We have added in the abstract and methods that participants included in the consortia were of European ancestry. Unfortunately, other demographic information about participants of all studies included in each consortium are poorly reported in the corresponding GWAS paper. Each of the four consortia include a very large number of separate cohort and case-control studies.**

9. Abstract: Methods and Findings: Please clarify that the associations between smoking and prostate cancer in the UK Biobank, and between overall cancer and alcohol consumption did not reach statistical significance.  Please provide the confidence intervals and p-values for: “A positive association between alcohol consumption and lung cancer was observed in the International Lung Cancer Consortium, but not in UK Biobank.”

**Response: As recommended, we have clarified that the associations between smoking and prostate cancer in UK Biobank, and between overall cancer and alcohol consumption did not reach statistical significance. We have provided the ORs, CIs and p-values in the sentence about alcohol consumption and lung cancer. The revised sentence is: “Genetically-predicted alcohol consumption was statistically significantly associated with lung cancer in the International Lung Cancer Consortium (OR 1.94; 95% CI 1.41-2.68; p=4.68×10-5) but not in UK Biobank (OR 1.12; 95% CI 0.65-1.93; p=0.686).”**

10. Abstract: Methods and Findings: Please revise this sentence to reflect that there was no statistically significant relationship between alcohol consumption and overall cancer, i.e. “no evidence” rather than “limited evidence”: “We found limited evidence that genetically-predicted alcohol consumption was associated with overall cancer (n=75 037 cases; OR 0.95; 95% CI 0.84-1.07; p=0.376).”

**Response: We have amended the sentence to: “We found no statistically significant association between genetically-predicted alcohol consumption and overall cancer (n=75 037 cases; OR 0.95; 95% CI 0.84-1.07; p=0.376).”**

11. Abstract: Methods and Findings: In the last sentence of the Abstract Methods and Findings section, please describe the main limitation(s) of the study's methodology.

**Response: We have added the following sentence: “The main limitation of this study is that precision was low in some analyses, particularly for analyses of alcohol consumption and site-specific cancers.”**

12. Abstract: Conclusions: Please revise the first sentence to: “Our findings support the well-established relationship between smoking and lung cancer... and suggest that smoking may also be a risk factor for cancer of the head and neck, oesophagus, stomach, cervix and bladder.”  Please revise the final sentence to: “We found no evidence supporting a relationship between alcohol consumption and overall or site-specific cancer risk.” or similar, to clarify the meaning of “an association...cannot be precluded.” (please also revise this in the final “Conclusions” paragraph of the Discussion section).

**Response: We have amended the conclusion paragraph of the abstract as suggested.**

13. Author Summary: Under “What did the researchers do and find?”: Please remove “strong or suggestive” from the first bullet point.

**Response: As suggested, we have removed “strong or suggestive” from that bullet point as well as throughout the manuscript.**

14. Author Summary: Under “What did the researchers do and find?”: Please remove “though
several estimates were in the same direction as those reported by observational studies.” as the directions aren’t specifically mentioned, this is not helpful information.

**Response: We have removed that text.**

15. Author Summary: Under “What do these findings mean?”: This text should be distinct from the scientific abstract. (Please see our author guidelines for more information:

<https://eur01.safelinks.protection.outlook.com/?url=https%3A%2F%2Fjournals.plos.org%2Fplosmedicine%2Fs%2Frevising-your-manuscript%23loc-author-summary&amp;data=02%7C01%7Csusanna.larsson%40ki.se%7C9dff4e6c21314132177708d7fb574b83%7Cbff7eef1cf4b4f32be3da1dda043c05d%7C0%7C0%7C637254224661331166&amp;sdata=fhtrNF%2BXebdrutqFxwmwnnf%2BgRir8cQu4ln1KxOprxM%3D&amp;reserved=0>) 

Please revise the bullet points; we suggest; In this study, we observed a relationship between smoking and lung cancer, as well as evidence that smoking may also be a risk factor for cancer of the head and neck, oesophagus, stomach, cervix, and bladder.”  For the second bullet point, we suggest: “We found no evidence supporting a relationship between alcohol consumption and overall or site-specific cancer risk.” or similar, to clarify the meaning of “an association...cannot be precluded.”

R**esponse: We have revised the bullet points as suggested.**

16. Methods: “All participants provided informed consent.” Please specify whether consent was written or oral.

**Response: We have now clarified that the consent was written (page 7).**

17. Methods: Please provide summary demographic information (population and setting, years of inclusion) for the study participants, in the UK Biobank and GWAS consortia.

**Response: Summary demographic information (population and setting, years of inclusion) have been provided for study participants in UK Biobank (page 6, last paragraph). Little information about participants included in all the studies that contributed the data to the consortia were provided in the corresponding published GWAS paper and on the websites for each consortium. However, it is reported that participants were of European ancestry and came from European and North American countries and Australia. The lung cancer consortium included both women and men, whereas the breast and ovarian cancer consortia included women only and the prostate cancer consortium men only. This demographic information has been added to the Methods as follows (page 6): “All participants included in the consortia were of European ancestry and came from European and North American countries and Australia. The lung cancer consortium included both women and men, whereas the breast and ovarian cancer consortia included women only and the prostate cancer consortium men only.”**

18. Methods: Please add the following statement, or similar, to the Methods: "This study is reported as per the Strengthening the Reporting of Observational Studies in Epidemiology (STROBE) guideline (S1 Checklist)."

**Response: We have added that statement to the Methods (page 9, last sentence of the paragraph “Protocol”).**

19. Results: Paragraph 2 (and throughout): Please revise “strong or suggestive evidence” and “suggestive evidence” to be more clear- if statistical significance is intended, please indicate that. If clinical significance is intended, please remove the term because there is no clinical aspect in the study and so clinical significance cannot be addressed.

**Response: As recommended, in the Results section and throughout, we have amended “strong or suggestive evidence” and “suggestive evidence” to indicate whether the association was statistically significant or not. Because of these changes, we have also revised the following text in the Statistical analysis (page 8, last sentence): “Associations that were statistically significant at a Bonferroni corrected p-value below 0.0013 (correcting for 2 exposures and 19 outcomes) were considered strong evidence of associations. Associations with p-values between 0.05 and 0.0013 were regarded suggestive evidence of associations, requiring confirmation.” to “Associations were considered statistically significant at a Bonferroni corrected p-value below 0.0013 (correcting for 2 exposures and 19 outcomes).”**

20. Results: Top of page 10: Please remove the word “slightly” from the sentence: “Adjustment for genetic predisposition to smoking using multivariable Mendelian randomization slightly attenuated the association of genetically-predicted alcohol consumption with lung cancer (OR 1.75; 95% CI 1.23-2.49; p=0.002) (S5 Table).”

**Response: “slightly” has been removed from that sentence.**

21. Results: Where you describe relationships between alcohol consumption and site-specific cancers, please make it clear in the text whether these relationships were statistically significant or not.

**Response: We have now made it clear in the Results section which associations were statistically significant or not.**

22. Discussion: First sentence: Please avoid assertions of primacy ("We are the first....")  We suggest you temper this with the phrase “To our knowledge…” or similar.

**Response: We have removed “we are the first” from that sentence.**

23. Discussion: First paragraph: Please revise this sentence, to temper the causal implications: “... offering strong support of causation to previous observational studies.”  (e.g., "supporting a causal relationship as consistent with previous observational ..." might be helpful).

**Response: We have amended the text to “… supporting a causal relationship as consistent with previous observational studies.”**

24. Discussion: Please present and organize the Discussion as follows: a short, clear summary of the article's findings; what the study adds to existing research and where and why the results may differ from previous research; strengths and limitations of the study; implications and next steps for research, clinical practice, and/or public policy; one-paragraph conclusion.  Specifically, your discussion is missing a final paragraph reflecting on the study’s implications.

**Response: We have added a final paragraph reflecting the study’s implications. The following text has been added (page 17): “These analyses broaden the evidence base for the harmful effect of cigarette smoking, which has previously been demonstrated for most cardiovascular diseases [44,45], type 2 diabetes [46] and bone fracture [47]. Those findings along with results of the present study provide further evidential support for public health interventions to reduce cigarette smoking initiation in the population, and suggest that these strategies will have an important impact on lessening the burden of major diseases, and of cancer in particular.”**

25. Conclusion: Please revise this sentence to: “The results of this study support the well-established relationship between smoking and lung cancer, and suggest that smoking may also be a risk factor for cancer of the head and neck, oesophagus, stomach, cervix and bladder. We found no evidence in support of a relationship between alcohol consumption and overall cancer risk, but associations between alcohol consumption and risk of site-specific cancer should be further investigated.” or similar.  Note that this paragraph appears to be identical to the abstract conclusions and the Author Summary.

 **Response: We have revised the sentence as suggested.**

26. Figure 1: In the legend, the “2” in I2 should be superscript.

**Response: This has been corrected.**

--------------------------------------

**Comments from Reviewers:**

**Reviewer #1:**

Larsson et al. have satisfactorily addressed the majority of my comments.

The only comment that I don't think was fully addressed was Major Comment 4. I think the caveat that Larsson et al. responded with should be added to the manuscript (i.e. that Wald-type methods are unbiased under the null, and that the estimators do not reflect meaningful causal parameters).

Alex Cornish (ICR, London)

**Response: As recommended, we have added to the manuscript that MR analyses of binary exposures could be problematic and that the Wald-type methods are unbiased under the null. The following text has been added to the limitations (page 16):**

**“An additional limitation is that smoking initiation is a binary exposure. An MR estimate with a binary exposure and binary outcome is difficult to interpret as a specific causal effect [42]. Furthermore, the MR estimate cannot simply be compared with the association between self-reported smoking and cancer risk estimated by observational studies. However, even in this setting, MR estimates are unbiased under the null [43], and thus provide a valid test of the causal null hypothesis even if the estimates do not reflect meaningful causal parameters. Here, we expressed all estimates in terms of the association between genetically-predicted levels of smoking initiation and the outcome, rather than making claims about the numerical magnitude of the causal effect.”**

--------------------------------------

**Reviewer #2:**

The authors have adequately addressed my comments.

Kostas Tsilidis, Imperial College London and University of Ioannina

**Response: Thank you.**
